# Supplementary material for: YgfY Contributes to Stress Tolerance in Shewanella oneidensis Neither as an Antitoxin Nor as a Flavinylation Factor of Succinate Dehydrogenase
Source: Microorganisms. 2021 Nov 9;9(11):2316. doi: 10.3390/microorganisms9112316 (PMC8621075; doi:10.3390/microorganisms9112316)
Supplement: Supplementary file 1 [file microorganisms-09-02316-s001.zip › microorganisms-1433271-supplementary.pdf]

Table S1. Strains and plasmids used in this study.

	Description of strains and plasmids	Source
S. oneidensis		
MR-1	Wild-type	ATCC
Δ*ygfY*	MR-1 derivative with deletion of SO_1339	This study
Δ*ygfX*	MR-1 derivative with deletion of SO_1340	This study
Δ*ygfYX*	MR-1 derivative with double deletion of SO_1339 and SO_1340	This study
Δ*sdh*	MR-1 derivative with deletion of SO_1927, SO_1928 and SO_1929	This study
E. coli		
JM109	General cloning host for plasmid manipulation	Lab stock
WM3064	Auxotrophic to DAP; used for conjugation	[16]
MG1655	Wild type	ATCC
BL21	Expression host carrying the T7 RNA polymerase	Novagen
BTH101	F-, cya-99, araD139, galE15, galK16, rpsL1 (Str r), hsdR2, mcrA1, mcrB1.	Euromedex
**Plasmid**		
pRE112	Suicide vector, Cm^r	[15]
pRE-*ygfY*	pRE112 derivative to delete *ygfY*	This study
pRE-*ygfX*	pRE112 derivative to delete *ygfX*	This study
pRE-*sdh*	pRE112 derivative to delete *sirCD*	This study
pBBP1	Constitutive expression vector in *Shewanella*	This study
pBBP1-*ygfY*$^{G16R/E19A}$	pBBP1 derivative to expression ygfY	This study
pBBP1-*ygfY*	pBBP1 derivative to expression ygfY	This study
pKT25	C-terminal fusion of the target protein to the T25 fragment of adenylate cyclase	Euromedex
pKNT25	N-terminal fusion of the target protein to the T25 fragment	Euromedex
pUT18	N-terminal fusion of the target protein to the T18 fragment of adenylate cyclase	Euromedex
pUT18C	N-terminal fusion of the target protein to the T18 fragment	Euromedex
pKNT25-*ygfX*	pKNT25 derivative to express YgfX fused with T25 in its N-terminal	This study
pKT25-*ygfX*	pKT25 derivative to express YgfX fused with T25 in its C-terminal	This study
pUT18-*mreB*	pUT18 derivative to express MreB fused with T18 in its N-terminal	This study
pUT18C-*mreB*	pUCT18 derivative to express MreB fused with T18 in its C-terminal	This study
pUT18-*ftsZ*	pUT18 derivative to express FtsZ fused with T18 in its N-terminal	This study
pUT18C-*ftsZ*	pUT18 derivative to express FtsZ fused with T18 in its C-terminal	This study
pUT18-*ygfY*	pUT18 derivative to express YgfY fused with T18 in its N-terminal	This study
pUT18C-*ygfY*	pUCT18 derivative to express YgfY fused with T18 in its C-terminal	This study
pET-28a(+)	Expression vector in *E. coli* under induction of IPTG	Novagen
pBAD24	Expression vector in *E. coli* under induction of L-arabinose	[17]
pET-*ygfY*	pET-28a(+) derivative to express YgfY	This study
pBAD-*ygfX*	pBAD24 derivative to express YgfX	This study

Table S2. Primers used in this study

Primers	DNA sequence
pRE-*ygfY*-F1	tgaactgcatgaattcccggggagcaaggccaactattgatcc
pRE-*ygfY*-R1	agcggacccgagcaatattc
pRE-*ygfY*-F2	gaatattgctcgggtccgctcagatcctgagcttgcccgtat
pRE-*ygfY*-R2	cgatcccaagcttcttctagaatccgagcacaccgattagg
check-*ygfY*-F	gcgggtgagtcgcttaaatatc
check-*ygfY*-R	cgctaccgaccattttgcaa
pRE-*ygfX*-F1	tgaactgcatgaattcccgggttccacgctactcgcatcg
pRE-*ygfX*-R1	gcaagaccatgtcctcagcc
pRE-*ygfX*-F2	ggctgaggacatggtcttgccctaatcggtgtgctcggatt
pRE-*ygfX*-R2	cgatcccaagcttcttctagatcgccttgccagtaactcac
check-*ygfX*-F	gagcgaatgatccagcatcctg
check-*ygfX*-R	gcgtcatgactgacctacatgg
pRE-*ygfYX*-F1	tgaactgcatgaattcccggggggtgagtgtggcgtgagtt
pRE-*ygfYX*-R1	atccgagcacaccgattaggcaacgaaaggctggaacaaca
pRE-*ygfYX*-F2	cctaatcggtgtgctcggatt
pRE-*ygfYX*-R2	cgatcccaagcttcttctagatcgccttgccagtaactcac
check-*ygfYX*-F	ttgctctcggttgtgcctaat
check-*ygfYX*-R	tcgctgcttagaactcggtatc
pRE112-F	aatcgtgttgaggccaacgc
pRE112-R	ttcaggttcatcatgccgtctg
pBBP1-*ygfY*-F	tttggagatagagtatctagattggagttaatgaatattgctcggg
pBBP1-*ygfY*-R	atcgaattcctgcagcccgggctaaaactatggcgccgctc
check-pBBP1-F	atgctctgctataggtctgttttg
check-pBBP1-R	gtgagcggataacaatttcacac
pRE-*sdh*-F1	tgaactgcatgaattcccgggctttactttccttgtcgcagct
pRE-*sdh*-R1	tcagcggaggctaaagatgaat
pRE-*sdh*-F2	tcatctttagcctccgctgagcagccagctcgtgaacat
pRE-*sdh*-R2	cgatcccaagcttcttctagagccgcccttagaatatccagat
check-*sdh*-F	ctggcaactctatcgagtcgt
check-*sdh*-R	cgcttcaggcgcgtctttaga
pKNT25-*ygfX*-F	gaccatgattacgccaagcttggtcgtttttgtttgtgtttgc
pKNT25-*ygfX*-R	ggtacccggggatcctctagagtaatccgagcacaccgattag
pKT25-*ygfX*-F	ggctgcagggtcgactctagaggtcgtttttgtttgtgtttgc
pKT25-*ygfX*-R	ttagttacttaggtacccggggaatccgagcacaccgattag
pUT18-*mreB*-F	gaccatgattacgccaagcttgttcaagaagctgcgtggc
pUT18-*mreB*-R	ggtacccggggatcctctagagtttcttcggagaacagatcgc
pUT18C-*mreB*-F	ggaacgccactgcaggtcgactttcaagaagctgcgtggca
pUT18C-*mreB*-R	gagctcggtacccggggatcctcggtttcttcggagaacagat
pUT18-*ftsZ*-F	gaccatgattacgccaagcttgtttgagatcatggacactcactc
pUT18-*ftsZ*-R	ggtacccggggatcctctagagtgtcagcttgcttacgcaaa
pUT18C-*ftsZ*-F	ggaacgccactgcaggtcgacttttgagatcatggacactcactc
pUT18C-*ftsZ*-R	gagctcggtacccggggatcctcgtcagcttgcttacgcaa
pUT18-*ygfY*-F	gaccatgattacgccaagcttggagttaatgaatattgctcgggt
pUT18-*ygfY*-R	ggtacccggggatcctctagagttggcgccgctcgtc
pUT18C-*ygfY*-F	ggaacgccactgcaggtcgactgagttaatgaatattgctcgggtc
pUT18C-*ygfY*-R	gagctcggtacccggggatcctctggcgccgctcgt
check-pNT25-F	gcgcaacgcaattaatgtgag
check-pNT25-R	tggcgaaggaaaaaaacgcc
check-pKT25-F	gtcaaggtgatcggcaatgc
check-pKT25-R	ggcgaagaactccagcatga

check-pUT18-F	agcgcaacgcaattaatgtga
check-pUT18-R	attttcgtcgtatcggccac
check- pUT18C-F	ggaaaagcctgttcgacgatg
check- pUT18C-R	cgcttacagacaagctgtgac
pET-*ygfY*-F	ctgccgcgcggcagccatatgttggagttaatgaatattgctcggg
pET-*ygfY*-R	gtggtggtggtggtgctcgagctatggcgccgctcgtc
check- pET-F	catgagcccgaagtggcg
check- pET-R	agtgtagcggtcacgctg
pBAD-*ygfX*-F	gaattcaccatggtacccgggttggtcgtttttgtttgtgtttgc
pBAD-*ygfX*-R	tccgccaaaacagccaagcttcaaatccgagcacaccgattag
check-pBAD24-F	cactgcgtcttttactggctc
check-pBAD24-R	gcgttcaccgacaaacaacag
RT-16S-F	gtattcaccgtggcattctgat
RT-16S-R	cgtgctacaatggcgagtac
RT-0226-F	tcgtacatcggcggtgaagg
RT-0226-R	taacacgaccaccacggatcaa
RT-0232-F	gtggcggtggcgtaactttc
RT-0232-R	cgtaccagctcagacagaatgc
RT-0234-F	gcggatggtgagcgtcgtta
RT-0234-R	gcagtggcattgcgttaccg
RT-0240-F	cgccaagtgaaacacccaatct
RT-0240-R	gcttcgtcatgtgcatggatct
RT-1357-F	cattcgtttagctcgtggtggc
RT-1357-R	cgttacggctgtcagcaacaa
RT-4247-F	gcaagaagccgatggttggtaa
RT-4247-R	ggcacgaagttcagcaacaaca
RT-3980-F	gtcctgacgtgcctcgctta
RT-3980-R	ccacctttcgcccacttacct

Table S3. Genes differentially expressed and discussed in this study.

Locus tag	Gene	Log_2FC^a
	**Translation**	
SO_0220	*rplK*	−1.942166546
SO_0221	*rplA*	−2.049792715
SO_0222	*rplJ*	−1.600958625
SO_0223	*rplL*	−1.028621152
SO_0226	*rpsL*	−3.517487594
SO_0227	*rpsG*	−1.792918536
SO_0230	*rpsJ*	−2.190586402
SO_0231	*rplC*	−1.590129805
SO_0232	*rplD*	−3.436693728
SO_0233	*rplW*	−0.904612056
SO_0234	*rplB*	−2.885597056
SO_0235	*rpsS*	−0.151719993
SO_0236	*rplV*	−1.319492831
SO_0237	*rpsC*	−1.800592037
SO_0238	*rplP*	−2.052906829
SO_0239	*rpmC*	−2.072734307
SO_0240	*rpsQ*	−3.669224847
SO_0241	*rplN*	−1.837570793
SO_0242	*rplX*	−0.655077486
SO_0243	*rplE*	−0.799852018
SO_0244	*rpsN*	−1.217068952
SO_0245	*rpsH*	−1.435998834
SO_0255	*rpsD*	−0.124712594
SO_0257	*rplQ*	−2.786801737
SO_0007	*rpmH*	−2.222923507
SO_1207	*rpsO*	−0.988565105
SO_1357	*rpsP*	−3.457547484
SO_1360	*rplS*	−2.083647562
SO_1629	*rpsB*	−0.054292953
SO_2301	*rpmI*	−2.931052646
SO_2302	*rplT*	−2.540746334
SO_2402	*rpsA*	−0.775620622
SO_2780	*rpmF*	−Inf
SO_2112	*rplY*	−3.661559819
SO_3651	*rpmA*	−2.765186899
SO_3652	*rplU*	−2.443500718
SO_3927	*rplI*	−2.322583716
SO_3928	*rpsR*	−0.697311358
SO_3930	*rpsF*	−1.486817506
SO_3537	*rpsT*	−1.644519769
SO_3939	*rpsI*	−1.281579528
SO_3940	*rplM*	−1.86936537
SO_4120	*rpmE*	−2.567067813
SO_4246	*rpmG*	−3.660546991
SO_4247	*rpmB*	−3.679349642
SO_0228	*fusA*	−1.617400249
SO_0229	*tufa*	−1.26182418
SO_1630	*Tsf*	−1.035723907
SO_1204	*infB*	−2.025350479

**Transcription**		
SO_0224	*rpoB*	−1.295594742
SO_0225	*rpoA*	−1.791716277
SO_0256	*rpoC*	−1.375418571
SO_0360	*rpoZ*	−2.268383885
SO_1191	*greA*	−1.592524
SO_1342	*rpoE*	0.736553
SO_3210	*fliA*	0.2519989
SO_3551	*rpoD*	1.0957651
SO_3432	*rpoS*	1.8089838
SO_4583	*rpoH*	−0.244422251
**Cell wall synthesis and cell division**		
SO_4217	*ftsQ*	−1.275213
SO_4218	*murC*	−1.642149
SO_4219	*murG*	−1.727472
SO_4220	*ftsW*	−2.281396
SO_4221	*murD*	−1.764096
SO_4222	*mraY*	−1.344993
SO_4225	*ftsI*	−1.274609
**Nitrite reduction**		
SO_0045	*nsrR*	−1.435095152
SO_3980	*nrfA*	−1.193113409

[a]. FC indicates fold change of transcription in Δ*ygfY* compared with wild-type.

Figure S1

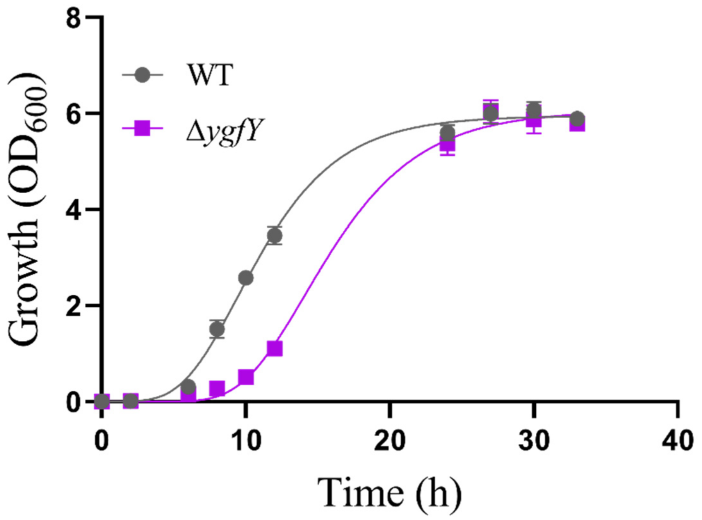

Figure S1. Growth of *ygfY* mutant and wild-type strains at 30 ℃. The data are the mean ± SD (n = 3).

Figure S2

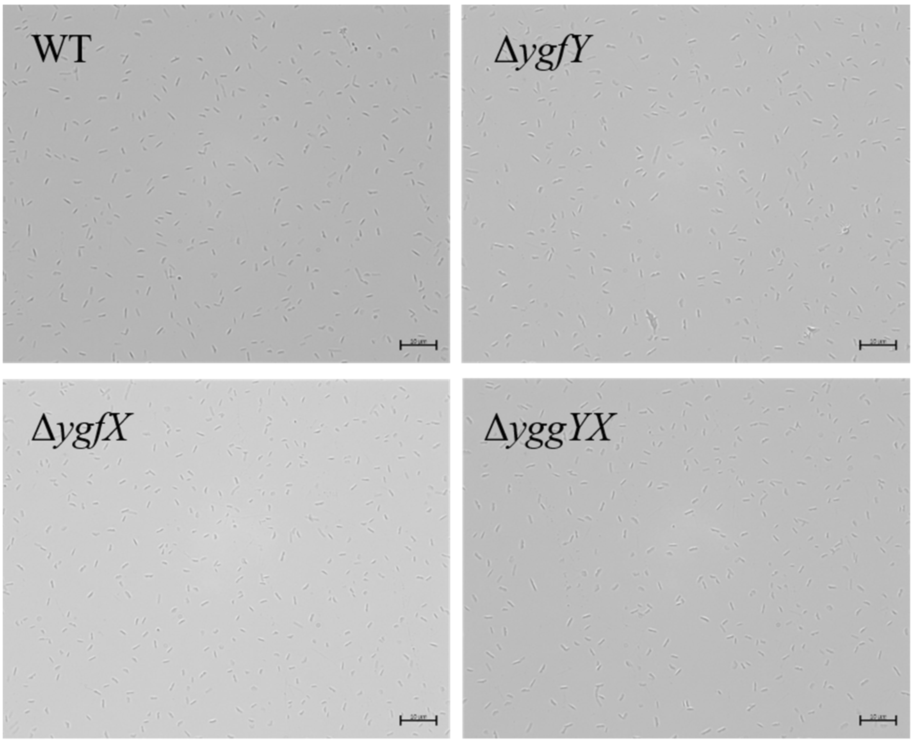

Figure S2. Cell morphology of Δ*ygfY*, Δ*ygfX*, Δ*ygfYX* and WT. Stains were cultured in LB at 16 ℃ for 48 h and subjected to observation under a microscope.

Figure S3

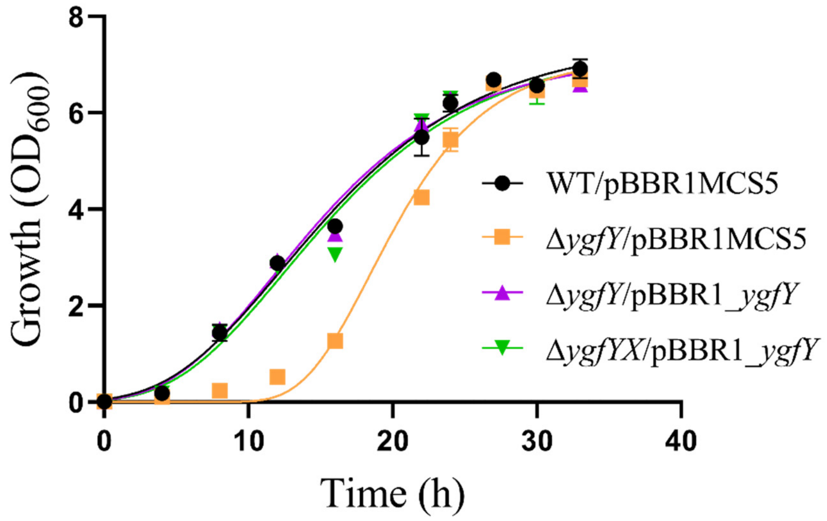

Figure S3. YgfY expressed from its native promoter revered the growth defect of Δ*ygfY*. Region containing *ygfY* coding sequence and 285 bp upstream was cloned into pBBP1MCS5 to express *ygfY* from its native promoter. Resulted plasmid pBBR1_*ygfY* was then transformed into strains of *Shewanella*. Strains transformed with the empty vector pBBP1MCS5 were set as controls. The data are the mean ± SD (*n* = 3).

Figure S4

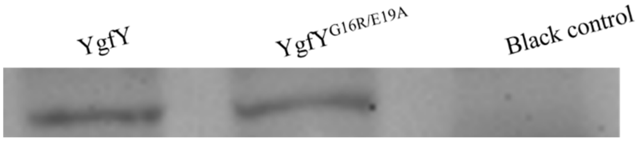

Figure S4. Expression of YgfY and YgfY$^{G16R/E19A}$. YgfY and YgfY$^{G16R/E19A}$ were fused with GST, and were expressed in Δ*ygfY* using pBBP1. Strains were cultured at 16 °C for 48 h, and collected for the analysis of western blotting. Blank control indicated sample of Δ*ygfY*/pBBP1.
